# Prenatal Exposure to Gossypol Impairs Corticogenesis of Mouse

**DOI:** 10.3389/fnins.2020.00318

**Published:** 2020-04-03

**Authors:** Xiaoyan Zhu, Yongji Wu, Cixia Li, Wenyong Yan, Jiarong Pan, Shuzhong Wang, Shanting Zhao

**Affiliations:** College of Veterinary Medicine, Northwest A&F University, Yangling, China

**Keywords:** gossypol, corticogenesis, neural progenitor cells, proliferation, differentiation

## Abstract

Gossypol is a yellow polyphenolic compounds extracted from roots, stems and seeds of cotton plants. Excessive intake of gossypol induces severe pathological signs of toxicity in livestock and wildlife. Currently, gossypol has received widespread attention for its toxic effects on the reproductive system. However, reports of the effects of gossypol during corticogenesis and the development of the mouse cerebral cortex are unavailable. In the present study, gossypol was orally administrated at a dose of 0, 20, and 50 mg/kg body weight/day to pregnant mice from embryonic day 6.5 to the time of sample collection. We used *in utero* electroporation and immunofluorescence to demonstrate that gossypol impaired cortical neuronal migration. Furthermore, labeling with 5-bromo-2-deoxyuridine and western blot analysis revealed that gossypol disturbed the balance between proliferation and differentiation of neural progenitors, inhibited neural progenitor cell proliferation, neuronal differentiation, and maturation. Additionally, cortical progenitor apoptotic cell death increased in the developing gossypol-treated cortex, which was associated with NF-κB and MAPK pathways. In conclusion, our findings indicate that gossypol exposure disrupted neurogenesis in the developing neocortex, suggesting the potentially harmful impact of gossypol on the cerebral cortex development of humans and livestock.

## Introduction

Cottonseed oil and cottonseed meal flour are by-products of cottonseeds and cotton plants commonly consumed by humans and food-producing animals as they are rich in oil and proteins (Huang et al., 2006; Camara et al., 2016). However, high concentrations of gossypol and its potential toxicity limit the efficient utilization of cottonseed and cotton plants (Chen et al., 2019). Gossypol, a yellow polyphenol compound, was initially investigated as a male contraceptive in China (Coutinho, 2002; Lopez et al., 2005). Previous studies have focused on the toxic effects of gossypol on the reproductive system, and immune function, while reducing resistance to infections, and impairing the efficiency of vaccines (Gadelha et al., 2014b). Cumulative evidence suggests that gossypol exhibits various pharmacological properties such as anti-inflammatory, anti-fungal, anti-fertility, and anti-cancer properties (Moon et al., 2011; Keshmiri-Neghab and Goliaei, 2014; Zeng et al., 2019). Molecular research shows that gossypol causes mitochondrial dysfunction by inhibiting cell respiration. Furthermore, it induces oxidative stress viewed as an imbalance between antioxidants and pro-oxidants, which results in the accumulation of reactive oxygen species (Kovacic, 2003; Keshmiri-Neghab and Goliaei, 2014; Santana et al., 2015). It has been reported that gossypol inhibits fast axonal transport and accumulates in the nerve, raising concerns about its possible neurotoxicity (Kanje et al., 1986). Therefore, the risk of gossypol toxicity has aroused worldwide attention due to the consumption of agricultural by-products such as cottonseed oil, milk, and meat from the affected animals.

The mammalian cerebral neocortex is a well-organized, six-layered structure composed of neurons. During cortical development, newborn neurons derive from the ventricular zone (VZ)/subventricular zone (SVZ) transition from multipolar to bipolar state and undergo radial glial-dependent migration to their final destination to form the cortical plate (CP) (Kriegstein and Noctor, 2004; Cooper, 2008; Frotscher, 2010). The development of the mammalian neocortex is indispensable to coordinate the proliferation, migration, and differentiation of neural progenitor cells (NPCs) (Florio and Huttner, 2014). It is widely believed that these processes are closely related to complex brain functions such as learning, memory, and cognition. The dysplasia of neocortex may lead to neurological disorders such as epilepsy, cognitive impairment, microcephalies, or hemimegalencephaly (Stouffer et al., 2016; Wang et al., 2017). However, the process of neurogenesis is regulated by intrinsic molecules, extrinsic signals and various environmental stimuli (Shimojo et al., 2008; Gotz et al., 2016). It has been reported that gossypol can cause hippocampal hemorrhage and changes in membrane permeability. It interferes with microtubule assembly, which may lead to the formation of apparent neurofibrillary tangles in rats (Sharma et al., 1966; Semon, 2012). Previous reports suggest that neurogenesis is regulated by a variety of pathological conditions that are commonly associated with microglial activation and inflammation in the brain, such as chronic stress and prenatal stimulation (Sierra et al., 2014). These findings may bring new ideas in investigating the neurotoxicity of gossypol in the central nervous system, especially during the development of the cerebral cortex.

The present study was undertaken to investigate the effect of gossypol exposure during embryonic neurogenesis in the developing neocortex. Here, we reported that gossypol exposure inhibited neuronal proliferation, differentiation, and maturation, as well as increased progenitor apoptotic cell death. Our results demonstrated the neurotoxicity of gossypol during corticogenesis and may help to understand the mechanism of gossypol neurotoxicity.

## Materials and Methods

### Reagents and Chemicals

Gossypol was purchased from Sigma-Aldrich (St. Louis, MO, United States). It was initially dissolved in a small volume of dimethyl sulfoxide (DMSO, Sigma-Aldrich, St. Louis, MO, United States), further diluted in 0.9% saline, and stored at −20°C. Working solutions of gossypol (0, 2.0, and 5.0 mg/ml) were freshly prepared every day. 5-Bromo-2′-deoxyuridine (BrdU) and propidium iodide (PI) were purchased from Sigma-Aldrich (St. Louis, MO, United States). 4′, 6′-diamidino-2-phenylindole (DAPI) was purchased from Invitrogen (Carlsbad, CA, United States). All other reagents and instruments used in this experiment are indicated below.

### Ethics Statement and Animals

All experimental procedures concerning animal care and handling were conducted according to the guidelines for Care and Experimental Use of Laboratory Animals of Northwest A&F University. All animal experiments were approved by the Animal Care Commission of the College of Veterinary Medicine, Northwest A&F University (certificate NO.: SCXK [SHAAN] 2017-003), in accordance with ARRIVE guidelines^1^.

Three months old male and female ICR mice were purchased from the Experimental Center of Xi’an Jiaotong University and adapted to the laboratory environment for 1 week. Mice were housed in a temperature-controlled room (22–26°C) on a light cycle (12 h light/12 h dark; light on from 8 a.m to 8 p.m), with *ad libitum* access to food and water. The time of vaginal plug appearance was designated as embryonic day (E) 0.5. Each dam was housed individually during the experiment.

### Drug Administration

Pregnant mice were randomly and assigned to three groups of five dams, and the investigators were blinded to study conditions. Pregnant mice received gossypol orally at a dose of 0, 20, and 50 mg/kg body weight/day from E6.5 to the time of sample collection. These doses were determined based on previous studies (Hahn et al., 1981; Li et al., 1989) and a preliminary study. A high dose was determined as the point when gossypol significantly altered neuronal migration of fetal cerebral cortex, but did not affect the maintenance of pregnancy and delivery of dams. Only one pregnant mouse was placed in each cage to prevent overcrowding. The number of pups per little was culled to 10 at postpartum day (P) 0. In every experiment, for one dosage group, newborn pups were selected randomly from five dams with five offspring in each group.

### Open Field Test (OFT)

**FIGURE 1:**
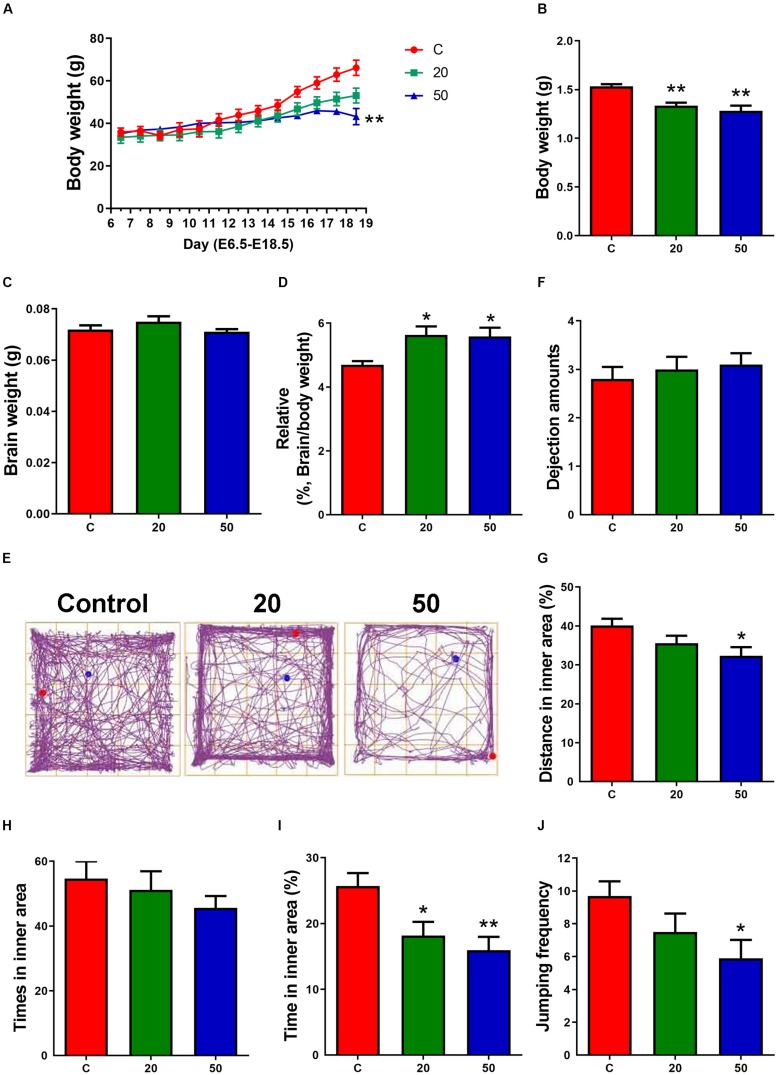
Exposure to gossypol suppressed the body weight of dams, body weights and the ration of brain weight to body weight of offspring. **(A)** Body weight of dams during E6.5 to E18.5. **(B)** Body weights of offspring at E18.5. **(C)** Brain weight of offspring at E18.5. **(D)** The ratio of brain weight to body weight of offspring at E18.5. **(A–D)** *n* = 5 per group. **(E)** Trace chart of open field test at P21 of offspring. The blue point represents the initial position, and the red point represents the end position. **(F–J)** Quantitative analysis of dejection amounts, distance, times, time traveled in the inner area, and jumping frequency. *n* = 10 per group. Difference was found between the control group and gossypol-treated groups. Results are presented as the mean ± SEM. *p < 0.05 and **p < 0.01 compared with the control group. E, embryonic day.

Open field test (OFT) measures the exploratory activity and anxiety behavior of mice in a novel environment (Hei et al., 2019). The mice at P21 were placed individually in the center of an open field box (50 × 50 × 30 cm) with inner and external areas. The box was divided into 25 equal squares including 9 cells in the center and the remaining 16 cells in the outer area (Figure 1E). Each session lasted 15 min, and dejection amounts, distance, times, proportion of the time traveled in the inner area, and jumping frequency were recorded immediately by using a video tracking/computer-digitizing system (ANY-maze) after the mice were put in the box. The box was wiped with 75% ethanol every time after each trail to avoid the influence of the residual odor on the experiment.

### *In Utero* Electroporation (IUE)

The plasmid expressing enhanced green fluorescence protein (*pCAG-GFP*) was purified using the E.Z.N.A.TM Plasmid Maxi Kit (Omega, United States) according to the manufacturer’s protocol. The *in utero* electroporation (IUE) procedure was performed as previously described (Li et al., 2018). The pregnant mice with embryos at E15.5 were anesthetized with amobarbital sodium (Sigma), followed by exposure of the uterine horns. Approximately 1.5 μl of a 3.0 μg/μl plasmid containing Fast Green was injected into the lateral ventricle of the embryonic brain with a fine pre-pulled glass micropipette. Electroporation was performed with a BTX electroporator (30 V, 50 ms, five times, 950 ms interval). Subsequently, the uterine horns were repositioned into the abdominal cavity. Following the recovery period, pups were collected and post-fixed with 4% paraformaldehyde (PFA) in 0.1 M phosphate buffer (PB; pH 7.4) at 4°C.

### BrdU Labeling

BrdU, a marker of neurogenesis, is incorporated into the DNA of dividing cells during the S-phase of the mitotic process (Kee et al., 2002). BrdU was dissolved freshly in 0.9% saline before use. To assess the cell proliferation, pregnant mice were received a single intraperitoneal injection of BrdU (50 mg/kg body weight) 2 h before sacrificed at E15.5. To examine the proliferation, differentiation and maturation of cells, pregnant mice were given intraperitoneal injection of BrdU at E15.5 and sacrificed at E16.5, E17.5, or E18.5. Brains were dissected at the different stages indicated and post-fixed with 4% PFA in 0.1 M PB (pH 7.4) at 4°C.

### Immunofluorescence Analysis

Brain sections were sliced into 60-μm thick coronal sections using a vibratome (VT 1000S, Leica, Germany). The sections were rinsed three times in 0.1 M PB (pH 7.4) and incubated overnight at 4°C with primary antibodies diluted in blocking solution (4% BSA, 1% normal goat serum and 0.3% Triton X-100 in PB). Sections were rinsed in 0.1 M PB and incubated for 3 h at room temperature with adequate fluorescent secondary antibodies. After three consecutive washes in 0.1 M PB, the sections were counterstained for 15 min in 0.1 μg/ml DAPI or PI. The slices were washed in 0.1 M PB, dried, and coverslipped using Fluoromount G (Southern Biotechnology). Negative controls were processed using 1% normal goat serum instead of primary antibody to show the specificity of the immunostaining. Immunofluorescence labeling was visualized using a structured illumination microscope (Zeiss observer Z1) or a confocal laser scanning microscope (TCS SP8, Leica, Germany).

Immunofluorescent detection of BrdU was performed as described previously (Li et al., 2017; Xu et al., 2018). Brain sections were denatured in 2 M HCl at 37°C for 30 min, rinsed with 0.1 M PB, neutralized in 0.1 M borate buffer (pH 8.5) for 30 min, and rinsed with 0.1 M PB, before incubation with primary antibodies.

The following primary antibodies were used in this study: rabbit anti-GFP (1:1000, Invitrogen, Carlsbad, CA, United States); rat anti-BrdU (1:500, Millipore, Temecula, CA, United States); rabbit anti-Ki67 (1:500, Abcam, Cambridge, United Kingdom); rabbit anti-p-histone H3 (1:500, Millipore, Temecula, CA, United States); mouse anti-Nestin (1:500, Invitrogen, Carlsbad, CA, United States); mouse anti-Tuj1 (1:500, Chemicon, Nuremberg, Germany); goat anti-Brn2 (1:300, Santa Cruz Biotechnology, United States). The following secondary antibodies were used: Alexa Fluor 647 goat anti-rat IgG (1:500, Chemicon, Nuremberg, Germany); Alexa Fluor 488 donkey anti-rabbit IgG (1:500, Invitrogen, Carlsbad, CA, United States); Alexa Fluor 568 donkey anti-goat IgG (1:500, Cell Signaling Technology, Boston, MA, United States); Alexa Fluor 555 goat anti-mouse IgG (1:500, Abcam, Cambridge, United Kingdom).

### Western Blot Analysis

Western blot analysis was performed as described previously (Li et al., 2018; Hei et al., 2019). In brief, the E16.5 and E18.5 neocortices of pups were isolated and lysed in ice-cold RIPA buffer (Solarbio, Beijing, China) containing 1 mM PMSF (Solarbio, Beijing, China). 10% acrylamide gels (SDS-PAGE) with an equal amount of 20 μg protein load in each lane were electrophoresed, and the proteins were transferred onto a 0.45 μm polyvinylidene difluoride (PVDF) membrane (Millipore, Massachusetts, United States). After blocking with 5% non-fat milk for 2 h at room temperature, the membranes were incubated overnight at 4°C with specific primary antibodies against p-histone H3 (1:1000, Millipore, Temecula, CA, United States), Nestin (1:1000, Invitrogen, Carlsbad, CA, United States), Tuj1 (1:1000, Sigma-Aldrich, St. Louis, MO, United States), Brn2 (1:1000, Santa Cruz Biotechnology, United States), nuclear transcription factor-kappa B (NF-κB), caspase-3, p44/42 MAP kinase (ERK1/2), phospho-p44/42 MAP Kinase (p-ERK1/2), p38, phospho-p38 (p-p38), and β-actin (1:1000, Cell signaling Technology, Danvers, MA, United States). After rinsing three times, the membranes were incubated with horseradish peroxidase (HRP)-conjugated goat anti-rabbit IgG antibody, donkey anti-goat IgG antibody, or goat anti-mouse IgG HRP linked antibody (1:5000, Cell signaling Technology, Danvers, MA, United States). The protein bands were detected by ECL plus (GE Healthcare, Buckinghamshire, United Kingdom) using the GelDoc XR Gel Documentation System (Bio-Rad). The band intensities were analyzed using ImageJ analysis software.

### Statistical Analysis

All statistical analyses were performed with GraphPad Prism 7 (GraphPad Software Inc., United States). Data were expressed as mean ± standard error of the mean (SEM), and n referred to the number of animals per experimental group. Statistical tests included one-way ANOVA followed by Tukey’s multiple comparisons test between groups. Statistical significance was set at *p* < 0.05.

## Results

### Reduced Body Weight and Induced Depression-Like Behaviors in Gossypol-Treated Offspring

The dams treated with gossypol and the control group mice had indistinguishable weights from E6.5 to E14.5 of the gestational period. Later, the weight of the gossypol-treated dams displayed a slow-growth trend compared to the controls, and a significant difference in body weight was observed at E18.5 (Figure 1A). The body weight of mice offspring in the gossypol-treated group (20 and 50 mg/kg) at E18.5 was significantly lower than that in the control group (Figure 1B). In contrast, the brain weights of offspring were not significantly different between gossypol-exposed and control groups (Figure 1C), while the ratio of brain weight of the offspring mice showed a significant difference between the control group and the group treated with gossypol (Figure 1D). Moreover, we investigated the behavior of offspring regarding brain function at P21 through open field test (Figure 1E). The gossypol-treated offspring showed an obvious reduction in the distance (Figure 1G) and time (Figure 1I) in the inner area, and jumping frequency (Figure 1J) in the box, and no differences were observed in the dejection amounts (Figure 1F) and times in inner area (Figure 1H). The results indicated anxiety- and depression-like behaviors of brain in the gossypol-treated offspring.

### Gossypol Inhibited Neuronal Migration

**FIGURE 2:**
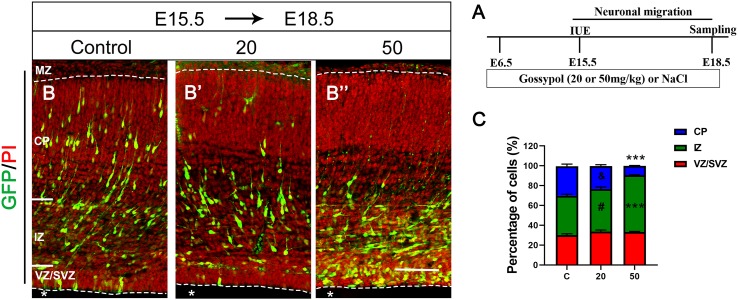
Exposure to gossypol inhibited neuronal migration in the developing cerebral cortex. Brain were electroporated with GFP (green) at E15.5 and fixed at E18.5 after IUE. Sections were counterstained with PI (red). **(A)** Protocol of IUE for testing the neuronal migration of cerebral cortex. **(B)** Representative immunofluorescence images of the GFP^+^ migrating neurons in the developmental cerebral cortex. **(C)** Distribution of GFP^+^ cells in each layer of cerebral cortex. Quantification of the number of neurons showed that, in control slices, one third neurons invaded the CP, whereas a large number of neurons accumulated in the IZ in the exposure gossypol groups. Results are presented as the mean ± SEM (*n* = 5 per group). Scale bars 100 μm. ***p < 0.001 compared with the control group; & and # showed no significant difference but has the trends compared with the control group. IUE, *in utero* electroporation; E, embryo; GFP, green fluorescent protein; MZ, marginal zone; CP, cortical plate; IZ, intermediate zone. VZ/SVZ, ventricular zone/subventricular zone. Asterisks indicate the ventricular lumen. Dashed lines indicate cortex boundaries.

To investigate the role of gossypol in the developing brain specifically during the period of prominent neurogenesis, we orally administered different concentrations of gossypol to time-pregnant mice from E6.5, injected *pCAG-GFP* at E15.5 using IUE, and performed histological analysis 3 days after electroporation (E18.5) to determine the neuronal migration (Figures 2A,B,B’,B”). We found that about 39.8% of GFP-positive cells had migrated into the intermediate zone (IZ). In contrast, numerous (43.0 and 57.1%) GFP-positive cells were found in the IZ of gossypol groups, at respective doses of 20 and 50 mg/kg. Small percentage (9.5%) of neurons labeled with GFP migrated into the CP in the group dosed with gossypol at 50 mg/kg, while the percentage of neurons was significantly higher in the control group (30.2%) (Figure 2C). These results suggested that gossypol treatment inhibited neuronal migration in the mouse cerebral cortex.

### Gossypol Impaired the Balance Between Proliferation and Differentiation of Neural Progenitor Cells

**FIGURE 3:**
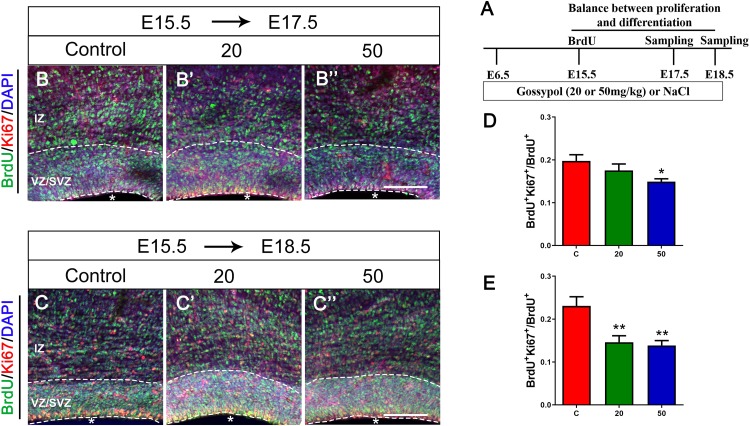
Exposure to gossypol break the balance between proliferation and differentiation of neural progenitor cells. A single pulse of BrdU was injected at E15.5 and fixed at E17.5 and E18.5. The brain slices were immunostained with antibody against BrdU (green), Ki67 (red), and counterstained with DAPI (blue). **(A)** Protocol of BrdU injections for testing the balance between proliferation and differentiation of neural progenitor cells. **(B)** Representative immunofluorescence images of the BrdU^+^ and Ki67^+^ cells at E17.5 in the developmental cerebral cortex. **(C)** Representative immunofluorescence images of the BrdU^+^ and Ki67^+^ cells at E18.5 in the developmental cerebral cortex. **(D)** Ratio of BrdU and Ki67 co-labeled neurons at E17.5 in the VZ/SVZ. **(E)** Ratio of BrdU and Ki67 co-labeled neurons at E18.5 in the VZ/SVZ. Exposure to gossypol caused a decrease in Ki67 incorporation in BrdU cells. Results are presented as the mean ± SEM (*n* = 5 per group). Scale bars 100 μm. *p < 0.05 and **p < 0.01 compared with the control group. E, embryo; IZ, intermediate zone; VZ/SVZ, ventricular zone/subventricular zone. Asterisks indicate the ventricular lumen. Dashed lines indicate cortex boundaries.

The balance between NPCs proliferation and differentiation is of critical importance in the development of cortical cortex. To investigate the effect of gossypol on NPCs, we injected BrdU at E15.5, and performed immunofluorescent staining at E17.5 and E18.5, respectively (Figure 3A). The brain slices were immunostained with antibodies against BrdU and proliferative marker Ki67, a nuclear protein expressed in all proliferating cells (Figures 3B,B’,B”,C,C’,C”). Statistical analysis showed that 14.92% of the cells in the gossypol 50 mg/kg group were BrdU^+^ Ki67^+^ double-labeled cells at E17.5, which was a significant reduction when compared to controls with almost 19.76% of co-labeled cells in the VZ/SVZ (Figure 3D). Similarly, this difference remained significant at E18.5. In the control group, 23.08% of BrdU^+^ Ki67^+^ double-labeled cells were present in the VZ/SVZ. In contrast, the co-expression neurons significantly reduced to 14.61 and 13.86% in the gossypol groups, at respective doses of 20 and 50 mg/kg (Figure 3E). This result suggested that gossypol may influence proliferating cells or their progeny by promoting cell exit from the germ zone of neocortex.

### Reduction in the Proliferation of Neural Progenitor Cells of Gossypol-Treated Mice

**FIGURE 4:**
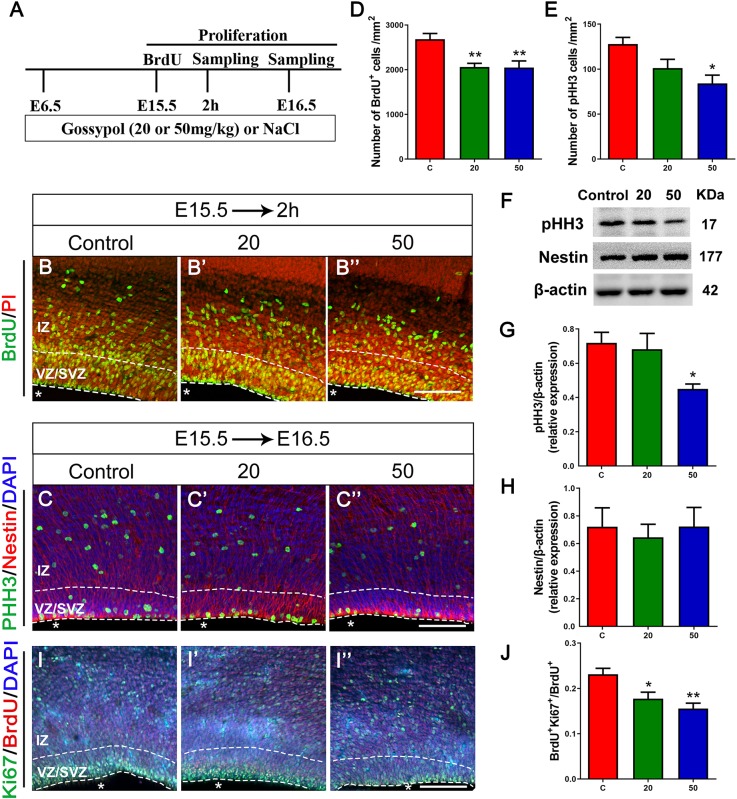
Exposure to gossypol inhibited proliferation of neural progenitor cells. **(A)** Protocol of BrdU injections for testing the proliferation of neural progenitor cells. **(B)** Representative immunofluorescence images of the BrdU^+^ cells at E15.5 after 2 h BrdU injection in the developmental cerebral cortex. The brain slices were immunostained with antibody against BrdU (green) and counterstained with PI (red). **(C)** Representative immunofluorescence images of the pHH3^+^ cells at E16.5 in the developmental cerebral cortex. The brain slices were immunostained with antibody against pHH3 (green) and Nestin (red), and counterstained with DAPI (blue). **(D)** Quantification of BrdU labeling cells. **(E)** Quantification of pHH3-positive cells. **(F)** Representative Western blots of pHH3, Nestin and β-actin are shown and densitometry was used to quantify the protein levels in the developmental cerebral cortex. **(G,H)** Relative protein levels of pHH3 **(G)** and Nestin **(H)** at E16.5, the expression of pHH3 was decreased after the treatment of gossypol. **(I)** Representative immunofluorescence images of the Ki67^+^ cells at E16.5 in the developmental cerebral cortex. The brain slices were immunostained with antibody against Ki67 (green), BrdU (red), and counterstained with DAPI (blue). **(J)** Quantification of Ki67-positive cells. Quantification of the number of neurons showed that, the number of BrdU^+^ cells, pHH3^+^ cells and the ratio of BrdU and Ki67 co-labeled neurons were significantly decreased in the slices of exposure to gossypol when compared with the control group. Results are presented as the mean ± SEM (*n* = 5 per group). Scale bars 100 μm. *p < 0.05 and **p < 0.01 compared with the control group. E, embryo; IZ, intermediate zone; VZ/SVZ, ventricular zone/subventricular zone. Asterisks indicate the ventricular lumen. Dashed lines indicate cortex boundaries.

As the VZ and SVZ are the proliferation zones of the cerebral cortex, we analyzed the number of proliferating NPCs to examine the inhibitory effect of gossypol on neuronal proliferation. E15.5 embryos were incorporated with BrdU 2 h before harvesting (Figures 4A,B,B’,B”). Statistical analysis showed a significant difference in the number of BrdU^+^ cells between the gossypol-treated and control groups (Figure 4D). To confirm the loss of progenitors in the gossypol treated groups, we performed pHH3, Nestin and Ki67 immunostaining in the VZ/SVZ. pHH3 is a marker for mitotic cells. We found that the number of pHH3^+^ cells had a decreasing dose-dependent trend following exposure to gossypol, which was significantly lower in the 50 mg/kg group than in the control group (Figure 4E). Nestin, a characteristic marker for NPCs, is widely expressed in the cerebral cortex and can mark the fibrous protuberance of NPCs. The results showed no significant differences in the area, sparse distribution, and arrangement of Nestin positive cells between gossypol-exposure and control groups (Figures 4C,C’,C”). Consistent with these results, we also found that the protein level of pHH3 was decreased following gossypol-treated group at a dose of 50 mg/kg compared to the control (Figures 4F,G), and no difference was observed in the expression of Nestin between gossypol-treated and control cortices (Figures 4F,H). To understand the link between the loss of proliferating progenitors in gossypol-treated mice and changes in the mode of cell differentiation, we injected BrdU into the abdominal cavity of pregnant mice on E15.5 and embryos were harvested 24 h after operation (E16.5) (Figure 4A). Cells that were co-labeled with BrdU^+^ and Ki67^+^ reentered the cell cycle. Consistent with the decreased numbers of BrdU^+^ and pHH3^+^ cells, the analysis revealed a significant decrease in the ratio of BrdU^+^ Ki67^+^ double-labeled cells over total BrdU^+^ cells in the gossypol-treated group (Figures 4I,I’,I”,J). Therefore, we concluded that the reduced number of proliferating NPCs in the VZ/SVZ of gossypol-treated mice was a result of decreased production of mitotic cells and a reduced cell-cycle reentry.

### Inhibited Neuronal Differentiation and Maturation in Gossypol-Treated Cortex

**FIGURE 5:**
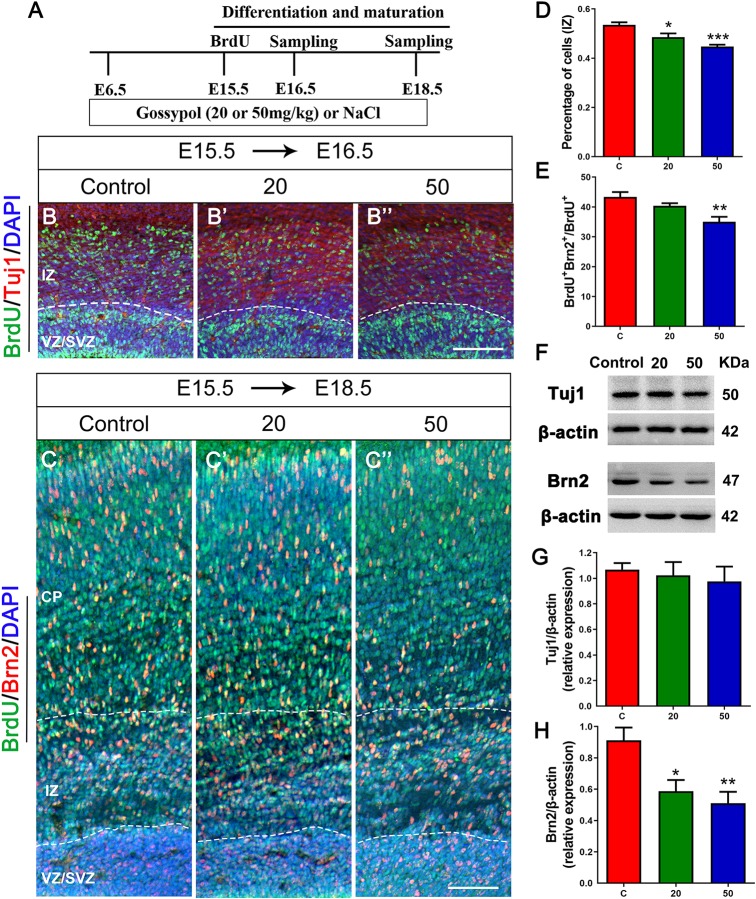
Exposure to gossypol inhibited neuronal differentiation and maturation. **(A)** Protocol of BrdU injections for testing the neuronal differentiation and maturation. **(B)** Representative immunofluorescence images of immunostaining for BrdU at E16.5. A single pulse of BrdU was injected at E15.5 and fixed at E16.5. The brain slices were immunostained with antibody against BrdU (green) and counterstained with DAPI (blue). Tuj1 (red) was used to label the IZ of the cerebral cortex. **(B)** Representative immunofluorescence images of the Brn2^+^ cells at E18.5. **(C)** A single pulse of BrdU was injected at E15.5 and fixed at E18.5. The brain slices were immunostained with antibody against BrdU (green) and Brn2 (red), and counterstained with DAPI (blue). **(D)** Quantification of BrdU labeling cells located in IZ. Less neurons entered the IZ in the gossypol exposure groups. **(E)** Quantification of BrdU^+^ Brn2^+^ positive cells in the total number of BrdU^+^ cells. Less cells differentiated into neurons at E18.5 in gossypol exposure groups. **(F)** Representative Western blots of Tuj1, Brn2 and β-actin are shown and densitometry was used to quantify the protein levels in the developmental cerebral cortex. **(G,H)** Relative protein levels of Tuj1 **(G)** at E16.5 and Brn2 **(H)** at E18.5, the expression of Brn2 was decreased after the treatment of gossypol. Results are presented as the mean ± SEM (*n* = 5 per group). Scale bars 100 μm. *p < 0.05, **p < 0.01 and ***p < 0.001 compared with the control group. E, embryo; CP, cortical plate; IZ, intermediate zone; VZ/SVZ, ventricular zone/subventricular zone. Dashed lines indicate cortex boundaries.

To further confirm if gossypol could influence neuronal differentiation, we injected BrdU at E15.5 and cortical slices were stained with Tuj1, a marker for young neurons (Figures 5A,B,B’,B”). The number of BrdU^+^ positive cells in Tuj1 positive cells located in the IZ was significantly lower in the gossypol-exposed groups at doses of 20 and 50 mg/kg than in the control group (Figure 5D). However, we studied the expression level of Tuj1 in cortex (Figure 5F) and found no significant difference between gossypol-exposed and control groups (Figure 5G). We performed Brn2^+^ immunostaining to examine the effect of gossypol on the maturation of neurons (Figures 5A,C,C’,C”). The analysis revealed a decreasing dose-dependent trend following gossypol exposure in the percentage of BrdU^+^ Brn2^+^ positive cells in total BrdU^+^ cells, which was significantly lower at a dose of 50 mg/kg than in the control group (Figure 5E). Moreover, significant decreases of Brn2 protein levels were observed when we performed western blot analysis for E18.5 gossypol-treated cortices (Figures 5F,H). Collectively, these results indicated that maternal gossypol exposure inhibited neuronal progenitor cells toward a neuronal fate and affected neuronal differentiation and maturation, which impeded neuronal development.

### Gossypol Induced Apoptotic Cell Death Associated With NF-κB and MAPK Pathway in Gossypol-Treated Cortex

**FIGURE 6:**
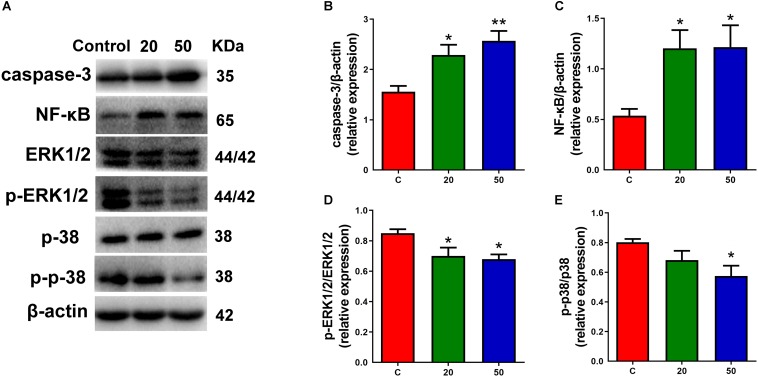
Exposure to gossypol induced progenitor apoptotic cell death. **(A)** Representative Western blots of caspase-3, NF-κB, ERK, p-ERK, p38, p-p38 and β-actin are shown and densitometry was used to quantify the protein levels in the developmental cerebral cortex. **(B,C)** Relative protein levels of caspase-3 and NF-κB, the expressions of caspase-3 and NF-κB were increased after the treatment of gossypol. **(D,E)** Ratio of phosphorylation of ERK and ERK, and ratio of phosphorylation of p38 and p38. Exposure to gossypol decreased the phosphorylation of ERK and p38. Results are presented as the mean ± SEM (*n* = 5 per group). *p < 0.05 and **p < 0.01 compared with the control group. p-ERK: phospho-ERK; p-p38: phospho-p38.

Multiple signals control the proliferation and differentiation of neural progenitor cells during corticogenesis. To understand the molecular mechanisms for gossypol regulated cortical neurogenesis, specifically the decreased neuronal maturation, we investigated the possibility of gossypol-induced apoptotic cell death in the neonatal cortex during the gestational period, using western blot analysis (Figure 6A). The protein levels of caspase-3, a marker of apoptosis, were examined. The results showed that the levels of caspase-3 significantly increased in gossypol-treated mice than in controls (Figure 6B). This suggested that the apoptotic cell death is likely a significant factor contributing to the reduced neuronal number in the gossypol-treated mice. Given the evidence that the NF-κB and mitogen-activated protein kinase (MAPK) signaling pathways play a crucial role in cell fate, the effects of gossypol on the activation of associated proteins were investigated (Figure 6A). The results showed that the protein levels of NF-κB significantly increased following gossypol exposure (Figure 6C). However, gossypol significantly decreased phosphorylation of ERK and p38 (Figures 6D,E). The results demonstrated that gossypol induced cortical progenitor apoptotic cell death and was possibly related to NF-κB and MAPK signaling.

## Discussion

Gossypol is slowly eliminated and tends to accumulate in the body (Sharma et al., 1966). It is known to reach the brain and bind randomly to vital cellular structures (Semon, 2012). Long-term feeding of cotton seed without gossypol detoxification or incomplete detoxification may result in slow growth, reduced reproductive efficiency, and death of livestock and poultry. The accumulation of gossypol in the human body may result in bleeding, loss of appetite, weight loss, infertility, hypokalemia, gastroenteritis, and neurological disorders. As the blood-brain barrier is not fully developed in the embryonic period, it provides limited protection against harmful substances entering the central nervous system. In the present study, we elucidated that exposure to gossypol during pregnancy disrupts neuronal migration, proliferation, and differentiation of offspring, which may be associated with progenitor apoptotic cell death. To our knowledge, this is the first report that demonstrates the direct effects of gossypol in mouse cerebral corticogenesis.

In the present study, decreased weight gain was observed in dams and offspring treated with gossypol. This finding is in agreement with Henry et al. (2001) indicating that the addition of gossypol to feed led to significant reductions in body weight and feed intake in chickens. Recent studies have also reported that reduced weight gain is a common sign of gossypol toxicity (Blevins et al., 2010; Zeng et al., 2014). Given that growth hormone plays a crucial role in growth and development, we suspect that these alterations may be related to the influence of gossypol on the regulation of the somatotropic axis in mice, and the decreased levels of growth hormone. However, the detailed mechanisms remain to be elucidated.

As the fetal thyroid gland produces inadequate amounts of thyroid hormone, the maternal thyroid supplies thyroxine in embryonic development. Gadelha et al. (2014a) reported that gossypol administered to rats was responsible for a significant reduction in serum thyroxine. Other studies showed the maternal thyroxine deficiency before the onset of fetal thyroid function (E17.5) affects reelin and its downstream signaling cascade, thereby response to aberrant neocortical neuronal migration and affecting neocortex development (Pathak et al., 2011; Chai et al., 2016). Our study showed that gossypol treatment inhibited neuronal migration in the cerebral cortex of embryonic mice. Although gossypol exposure may not influence the mature nervous system of dams, it may affect the development and function of the embryonic neocortex of the offspring by suppressing the maternal thyroid function. This observation explains, to some extent, that the nervous system of mammalian embryos is more sensitive than that of adults. It may be easily affected by neurotoxic exposure and lead to developmental disorders. This could be explained by the investigation of OFT in mice at P21. OFT is a classical method to evaluate anxiety-like and depression-like state in rodents. Mice treated with gossypol travelled less distance and spent less time in inner area, demonstrating the decline of curiosity and exploratory response in face of unfamiliar environment and increase of anxiety-like and depression-like behaviors.

The development of the mouse neocortex is a complex process initiated by the proliferation of neuronal precursors in the VZ, followed by neuronal differentiation and migration (Sidman et al., 1959). Recently, there have been considerable evidence to demonstrate that developing neocortex can be influenced by adverse effects, including prenatal stress and physical or chemical factor (s) and so on. Some researchers found that maternal exposure to bisphenol A affected neurogenesis in the developing neocortex and disrupted normal neocortical development by accelerating neuronal differentiation/migration (Nakamura et al., 2006; Komada et al., 2012). Other experiments revealed the impaired dendritic structure, cortical dysgenesis, and behavioral abnormalities following perinatal dioxin exposure (Haijima et al., 2010; Kakeyama et al., 2014; Kimura et al., 2015). In the present study, we found that gossypol disturbed the balance between proliferation and differentiation of NPCs. Although few BrdU^+^ cells were detected, the findings could not confirm that gossypol exposure inhibited the proliferation of NPCs. To provide additional support, we performed pHH3 immunofluorescent staining and western blot, found decreased numbers of pHH3^+^ cells and its expression in the gossypol group dosed at 50 mg/kg, indicating that gossypol exposure inhibited the mitotically active cells. Moreover, the cell cycle is a sequence of events by which a cell duplicates its genome, grows, and divides (Poon, 2016). In neural cells, proliferation and growth arrest are regulated by a balance of intrinsic and extrinsic factors that direct entry and exit from the cell cycle (Cunningham and Roussel, 2001; Dehay and Kennedy, 2007). Ki67 labels cells in the active phases of the cell cycle. Its expression within cells labeled with BrdU indicates that the cells are remaining in the active cell cycle (Wang et al., 2011). Previous study found significant increase of cell cycle exit in the bisphenol A treated mice (Komada et al., 2012). Interestingly, fewer cells remained in the active cell cycle in the gossypol-administrated group, suggesting that the inhibition of gossypol on NPCs cell cycle results not from remaining cells in the cell cycle, and rather from promoting cells to exit the cell cycle. The implication is that gossypol may shorten the duration of the cell cycle by inducing cell cycle exit. Thus, gossypol exposure inhibited neuronal proliferation, depending on the mitotic activity and the cell cycle of NPCs.

In addition to its inhibitory effect on NPCs proliferation, we demonstrated that gossypol could determine the fate of cortical neurons produced. The results showed that gossypol decreased the number of BrdU^+^ positive cells in Tuj1 positive cells located in the IZ. Thus, gossypol reduced NPCs by inhibiting their proliferation and neuronal differentiation. This phenotype may be caused by developmental delay following exposure to gossypol. Furthermore, Munekazu Komada et al. (2012) reported that drug exposure affected estrogen signaling, resulting in defective neuronal maturation and migration (Komada et al., 2012). Gossypol has been shown to affect estrogen production by specifically interacting with a nucleophilic site on rat alpha-fetoprotein that influences estrogen binding (Baker, 1984; Basini et al., 2009). Estrogens have neurotrophic and differentiation-promoting effects on neurons, which are critical during the period of brain development (Toran-Allerand et al., 2002). By co-labeling BrdU and Brn2, we observed a decrease of BrdU^+^Brn2^+^ mature cells following maternal exposure to gossypol at a dose of 50 mg/kg. These results presumably reflect that gossypol may influence estrogenic activity through down-regulation of estrogenic signaling target gene expression in the central nervous system. Therefore, future studies should examine the signaling pathways for a better understanding of the underlying mechanism of gossypol neurotoxicity.

We rationalized that gossypol induced a significant increase in the number of cortical progenitors exiting the cell cycle, thereby implying that the number of cells increased as they underwent differentiation. However, the mature neurons were significantly decreased on E18.5. Notably, gossypol increased caspase-3 levels, which revealed that gossypol-induced apoptotic cell death led to a decrease in mature neurons. It has been reported that NF-κB pathway activation stimulates cellular production of proinflammatory cytokines and apoptotic factors, resulting in neurotoxicity to the brain (Yepes et al., 2005). Previous study found that treatment with 10 μM gossypol induced apoptosis, and suppressed NF-κB activity and NF-κB-related gene expression in human leukemia U937 cells, which suggested that NF-κB is an essential target for the apoptotic effects of gossypol (Moon et al., 2008). However, in the present study, the influence of gossypol on NF-κB levels is of interest in further research as this transcription factor is activated following exposure to gossypol and contributes to neuronal apoptotic cell death. These disparate results can be shown that the role of NF-κB under gossypol treatment is diverse in different models. Furthermore, it should be noted that ERK and p38 have been associated with many cellular responses, including cell survival, proliferation, differentiation and apoptosis (Raman et al., 2007; Burmistrova et al., 2011). Gossypol decreased the phosphorylation of p38 and biphasic phosphorylation of ERK, suggesting that ERK and p38 may be involved in survival signals following gossypol exposure. Although the mechanisms by which gossypol decreased the phosphorylation of ERK and p38 are unknown, a possible explanation could be that the production of inflammatory cytokines and apoptotic factors downregulated ERK and p38 activities. Thus, apoptotic cell death and neurogenesis in the presence of maternal stimulation is still poorly understood and requires in-depth research.

## Conclusion

Using *in utero* electroporation and immunofluorescence analysis, the present study, for the first time, provided the evidence for the effects of maternal oral exposure to gossypol on neurogenesis in the developing neocortex, apart from its role in steroidogenesis. Maternal exposure to gossypol was associated with the disruption of neuronal migration. Notably, gossypol inhibited NPCs proliferation and neuronal differentiation. It disrupted the cell cycle, which consequently impeded neuronal development. Gossypol may be associated with increased progenitor apoptotic cell death related to NF-κB and MAPK pathways. These findings indicate that gossypol, as an agricultural by-product, is potentially hazardous to fetal development. This study may help to address a previously less-touched perspective of gossypol. Future studies on brain development following *in utero* exposure to gossypol are needed to evaluate the pathological changes in the cerebral cortex and elucidate the potential mechanism of gossypol neurotoxicity.

## Data Availability Statement

All datasets generated for this study are included in the article/supplementary material.

## Ethics Statement

All experimental procedures concerning animal care and handing were conducted according to the guide lines for Care and Experimental of Laboratory Animals of Northwest A&F University. All animal experiments were reviewed and approved by the Animal Care Commission of the College of Veterinary Medicine, Northwest A&F University (certificate NO.: SCXK [SHAAN] 2017-003) in accordance with ARRIVE guidelines (https://www.nc3rs.org.uk/arrive-guidelines).

## Author Contributions

The manuscript was written through contributions of all authors. All authors have given approval to the final version of the manuscript. XZ and SZ conceived the project idea, performed the data analysis, and finalization of the manuscript. XZ, YW, and CL performed majority of the experiments, participated in the discussion and analysis of data. WY, JP, and SW conducted parts of the experiments, and participated in discussion and analysis of the data. SZ supervised the project. XZ is the corresponding author and SZ is the co-corresponding author of this manuscript.

## Conflict of Interest

The authors declare that the research was conducted in the absence of any commercial or financial relationships that could be construed as a potential conflict of interest.

## Footnotes

1 https://www.nc3rs.org.uk/arrive-guidelines
